# Decision-making in borderline hip dysplasia and concomitant femoracetabular impingement syndrome: using a discrete choice experiment to explore patient preferences

**DOI:** 10.1093/jhps/hnae002

**Published:** 2024-02-01

**Authors:** Grant H Cabell, Nicholas F Kwon, Christopher Shultz, Carolyn A Hutyra, Brian D Lewis, Steven A Olson, Michael J Salata, Shane J Nho, Richard C Mather III

**Affiliations:** Department of Orthopaedic Surgery, Duke University Medical Center, 3475 Erwin Rd, Durham, NC 27705, USA; Department of Orthopaedic Surgery, Stanford Medicine, 450 Broadway, Redwood City, CA 94063, USA; Department of Orthopaedic Surgery, University of New Mexico, 2211 Lomas Blvd NE., Albuquerque, NM 87106, USA; Department of Orthopaedic Surgery, Duke University Medical Center, 3475 Erwin Rd, Durham, NC 27705, USA; Department of Orthopaedic Surgery, Duke University Medical Center, 3475 Erwin Rd, Durham, NC 27705, USA; Department of Orthopaedic Surgery, Duke University Medical Center, 3475 Erwin Rd, Durham, NC 27705, USA; Department of Orthopaedic Surgery, University Hospitals Case Medical Center, 11100 Euclid Ave, Cleveland, OH 44106, USA; Department of Orthopaedic Surgery, Rush University Medical Center, 1611 W Harrison St, Chicago, IL 60612, USA; Department of Orthopaedic Surgery, Duke University Medical Center, 3475 Erwin Rd, Durham, NC 27705, USA

## Abstract

Decision-making regarding surgical treatment of patients showing radiographic evidence of femoroacetabular impingement syndrome (FAIS) in the setting of borderline hip dysplasia (BHD) remains a challenge as there is no consensus on treatment in current literature. When medical evidence is unclear, understanding patient preferences becomes particularly important in deciding the optimal treatment for each patient. The purpose of this study was to measure the patient-determined importance of factors surrounding surgical treatment of FAIS in BHD. Patients aged 18–65 with hip pain and BHD (defined as lateral center edge angle 18–25 or Tonnis angle 10–15) morphology were given a discrete-choice experiment (DCE) focusing on attributes that differ between treatment options: Length of Hospital Stay, Major Complication Rate, Chance of Needing Reoperation within 2 Years and Time to Return to Regular Exercise. This DCE was used to calculate treatment preferences, relative attribute importance and preference weights. A total of 101 patients fully completed the DCE. The most important attribute (average importance weight, 95% CI) was Chance of Reoperation (60.16, 56.99–63.34), while the least important was Hospital Stay (6.57, 5.73–7.41). Only 6 Months to Resume Regular Exercise and 2% Chance of Reoperation (*P* < 0.05) significantly impacted treatment choice. When presented with fixed choice parameters, 50.5% of subjects preferred PAO and arthroscopy while 49.5% opted for arthroscopy alone. When no clear surgical treatment is indicated, patient preferences have an amplified role in patient decision-making. Our results confirm variation in attribute importance within treatments as well as treatment choice, highlighting the importance in understanding patient preferences in decision-making for FAIS in BHD. More patient-specific generalizable outcomes of surgical treatment options are needed in the literature.

## INTRODUCTION

The hip joint is an intricate structure in which stability is critical for proper function. Dynamic muscular stabilizers, static soft tissue supports and the labral suction seal all contribute to this stability [1–4]. Stability is also conferred by the acetabular orientation and its coverage of the femoral head [5–9]. In many patients, hip pathology is driven by abnormal bony morphology, namely Femoroacetabular Impingement Syndrome (FAIS) and hip dysplasia [5, 10, 11].

FAIS describes a pathological condition in which the bony morphology of the acetabulum and proximal femur can lead to repetitive microtrauma of the acetabular labrum and articular surfaces. This process can result in pain, instability and acceleration of progression to hip osteoarthritis [6, 12–14]. However, radiographic FAI morphology does not always occur in isolation; it has been found in 40–75% of patients with hip dysplasia [7, 15]. In hip dysplasia, insufficient acetabular coverage of the femoral head leads to greater reliance on the labrum and hip capsule to provide stability, which can result in increased instability and risk of progression to osteoarthritis [8, 13, 16–21]. Hip dysplasia is described on a spectrum, with terms such as mild, borderline, moderate and severe all used to characterize pathology.

Treatment of these pathologic morphologies varies depending on the driving source of symptoms. Periacetabular osteotomy (PAO) is the gold standard treatment for hip dysplasia (Lateral Central Edge Angle (LCEA) >25, Tonnis angle >10) reorienting the acetabulum to increase femoral head coverage [1, 22–30]. Studies have shown this method to be effective in improving hip function in both severe and borderline dysplastic cohorts [22, 24–27, 31–33]. However, given the increased morbidity associated with this invasive procedure, proper patient selection based on disease severity is critical. Arthroscopy alone has been shown to be an effective treatment to address intra-articular pathology by augmenting soft tissue stability through strategies such as labral repair and capsular plication, but it is limited in its utility to address significant acetabular pathologic morphology. Although arthroscopy is a soft tissue solution to an osseous abnormality, it can be an effective treatment in hip dysplasia morphology where symptoms do not seem to stem from instability [34]. As both are acceptable and well described treatment choices in borderline hip dysplasia (BHD) with FAIS, no consensus exists on the optimal surgical strategy for these patients.

**Fig. 1.**
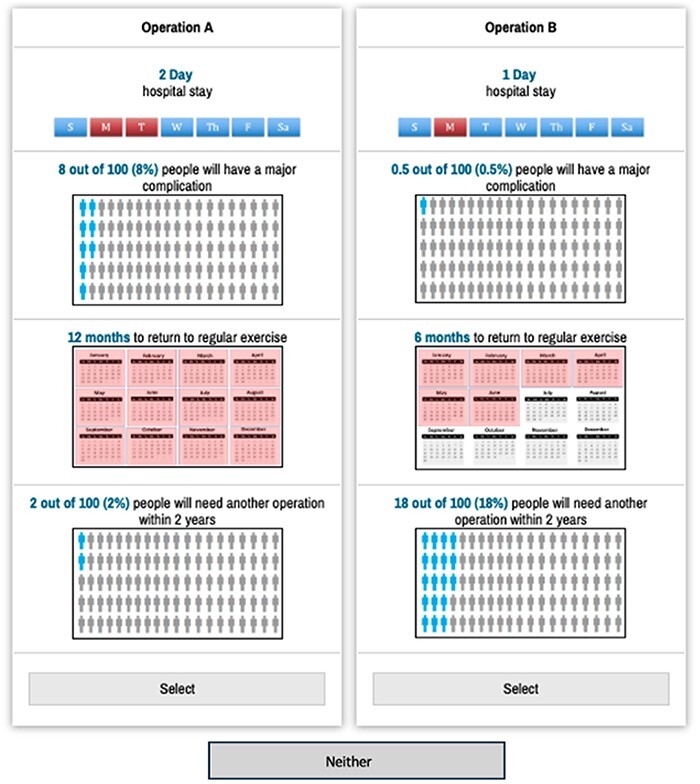
Shows an example of a discrete choice experiment question. Figure created in Lighthouse Studio from Sawtooth Software (Provo, UT, USA).

Due to lack of treatment consensus of FAIS in BHD, consideration of all factors including patient preferences is important in the shared decision-making (SDM) process. Characterization of patient preferences is an emerging tool with utility in guiding patients along a shared decision pathway; however, little literature exists on SDM in this context [35]. The purpose of this study was to use a discrete choice experiment (DCE) to investigate the relative importance patient’s assigned to different factors associated with surgical treatment of FAIS in the setting of BHD. Furthermore, we sought to explore if patients had similar preferences, and how to characterize these sets of patients into clinically meaningful groups.

## METHODS

Stated-preference methods have increasingly been applied to medical decision-making, which are particularly relevant when medical evidence does not suggest a clear treatment option. Using a DCE, we developed a survey following previously established good practice guidelines to quantify risk–benefit tradeoffs for surgical treatment of FAIS in the setting of BHD [36–40] (Fig. 1).

Survey length, attribute levels and understanding of attribute definitions were iteratively evaluated throughout the tool’s development with input from high volume hip preservation surgeons (six total, five both open and arthroscopic, one arthroscopic only) and patients. To assure objectivity, clinical information to populate the levels of the attributes was compiled by an independent reviewer. Seven individuals (four non-medical) pilot-tested the survey for assessment of clarity and cohesiveness; their feedback was used to optimize the survey.

**Table I.** Discrete choice experiment attributes and corresponding levels

*Attribute*	*Level*
Hospital stay	1 day
	2 day
	4 day
Major complication	0.5% complication risk
	2% complication risk
	8% complication risk
Months to resume regular exercise	3 months to resume
	6 months to resume
	12 months to resume
Chance of reoperation within 2 years	2% reoperation chance
	18% reoperation chance
	54% reoperation chance

Necessary approval by the Institutional Review Board (IRB) (Pro00067150) was received prior to data collection. In this cross-sectional study, participants aged 18–65 with hip pain and BHD morphology were recruited via telephone, email and from in-person visits to hip preservation clinic (one arthroscopic only, two open and arthroscopic surgeons) at a tertiary care academic medical center. While hip dysplasia is a spectrum of disease and understanding the limitations of using fewer parameters to classify patient pathology, we chose to define BHD as LCEA 18–25 or Tonnis angle 10–15 for this project consistent with existing literature [2, 41–46]. Patients were screened for radiographic evidence of FAIS as a prerequisite for scheduling in clinic. Additionally, senior authors reviewed radiographs of every patient in the study and confirmed clinical evidence of FAIS by clinical symptoms (such as hip pain with deep flexion, mechanical symptoms suggestive of a labral tear) and physical exam (limited internal rotation at 90° of hip flexion, pain with flexion-adduction-internal rotation of hip). Two hundred sixteen participants were deemed eligible. Incomplete responses (*N* = 42) were discarded, and a number of participants opted out due to survey length or time constraints surrounding their clinic visit. Only complete responses (*N* = 101) were analyzed. All participants gave informed consent prior to study enrollment. The study was supported internally with general institutional research funds.

All participants were asked to complete a survey consisting of demographic questions, educational material about FAIS and BHD, PAO and hip arthroscopy treatment and the DCE. The DCE included four attributes: length of hospital stay, risk of major complication (such as blood clot, infection and iatrogenic fracture), time to return to regular exercise and risk of needing as second operation within 2 years. Attributes and their respective levels were created via evidence published in the literature, review from stated preference experts who optimized levels for survey balance and input from fellowship trained hip preservation surgeons and patient interviews [23, 27, 47–51] (Table I). A short series of comprehension questions ensured participant understanding of relevant information to their decision.

**Table II.** Surgical treatment fixed choice parameters with final choice data

*Attribute*	*PAO and arthroscopy*	*Arthroscopy alone*
Months to resume normal exercise	12 months	6 months
Chance of reoperation	2%	18%
Hospital stay	4 days	1 day
Major complication rate	8%	0.5%
Final choice selection percentage (*n*)	50.5% (51)	49.5% (50)
Percent certainty for final choice	68.8% ± 23.0	

Participants completed 10 DCE trade-off questions exploring the importance of different attributes, followed by a final fixed trade-off question eliciting preference for PAO and arthroscopy or arthroscopy alone. Lighthouse Studio (Sawtooth Software, Provo, UT, USA) generated 216 different questionnaires that were randomly assigned to ensure objectivity and limit bias. These surveys presented the participant with the choice of either surgery A or surgery B with the same attributes, but the levels of the attributes were different between surveys. The DCE included connectivity and positional balance but did not include attribute randomization. Each random trade-off question also contained a third ‘opt out’ answer choice if patients wished to remain in their current functional state and not undergo surgery with the presented attributes.

Statistical analysis was completed using Lighthouse Studio and RStudio (Rstudio, Boston, MA, USA). Validity was ensured by two fixed attention checks, safeguards against straightlining (participants who always select option A or Option B) or not taking adequate time to complete the survey as these are strong indicators of lack of participant attention [52]. Only one respondent failed any of these three validation assessments; their responses were excluded from the final cohort.

Preference and importance weights were determined using Haversian Bayesian analysis. Importance weights measure the relative importance of an attribute, while preference weights are log-odds indicating average preference for specific levels within an attribute [35]. Notably, preference weights cannot be compared between attributes. Both intra-attribute preference weights and attribute importance weights were compared via unpaired *t*-tests. Separate Haversian Bayesian analysis was also performed with each demographic variable as a covariate, and a Tukey’s honestly different significance test with Bonferroni correction was used to confirm that no demographic variables may have influenced our results. A multivariate logistic regression was used to determine how individual levels of attributes influenced final surgical preference.

Maximum Acceptable Risk (MAR) was calculated for Chance of Reoperation and Months to Resume Regular Exercise to assess the additional risk (reoperation) that participants would be willing to incur for every additional unit of added benefit (months of quicker return to return to exercise). While MAR could be calculated for the levels between any two attributes, we specifically chose the parameters related to only hip arthroscopy surgery to provide the greatest clinical utility [23, 51, 53] (Table II). The MAR was calculated using Equation 1 below:


(1) $$MAR = \frac{{{\text{a}}/{\text{p}}}}{{{\text{b}}/{\text{q}}}}$$

Here, *a* was the preference weight for the 6 months return to regular exercise, *b* was the preference weight for 18% chance of reoperation within 2 years, both of which were obtained from our DCE data. *P* and *q* were 6 (months to return to regular exercise) and 18 (percent chance of reoperation in the next 2 years), respectively. These methods have been successfully used to calculate MAR in previous literature [54].

Latent class analysis was used to detect existing subgroups of participants with similar preferences in our sample [55]. Models with 2–5 subgroups were run, with log-likelihood (LL) and Bayesian Information Criterion (BIC) used to select the optimal number of subgroups as well as the mean preference and importance weights for each subgroup in the best fitting model.

## RESULTS

Two hundred and sixteen patients met our inclusion criteria, 101 of which fully completed the survey (46.8%). Of these participants, 75 (74.2%) were female, 81 (80.2%) were Caucasian, and the average age was 35.2 ± 10.7 years. Full demographics are found in Table III.

**Table III.** Demographics

*Respondent characteristics*	*Sample percentage (number)*
**Age**	35.2
**Sex**	
Male	25.8% (26)
Female	74.2% (75)
**Race**	
White	80.2% (81)
Black/African American	10.8% (11)
Hispanic or Latino	5.0% (5)
Asian or Pacific Islander	1.0% (1)
Native American or American Indian	2.0% (2)
Other/prefer not to identify	1.0% (1)
**Marital status**	
Single	37.6% (38)
Married	54.4% (55)
Separated or divorced	5.0% (5)
Widowed	0% (0)
Other	3.0% (3)
**Highest education level completed**	
Did not complete HS	2.0% (2)
High school/GED	3.0% (3)
Some college	29.7% (30)
Bachelor’s degree	33.7% (34)
Master’s degree	24.8% (25)
Advanced graduate work or PhD	6.9% (7)
**Employment**	
Employed full time (30+ h/week)	71.2% (72)
Employed part time (<30 h/week)	7.9% (8)
Not employed	18.8% (19)
Retired	2.0% (2)
**Household annual income**	
Less than 20 000	3.0% (3)
20 000–39 000	10.9% (11)
40 000–59 000	15.8% (16)
60 000–79 000	18.8% (19)
80 000–99 000	8.9% (9)
100 000 or more	37.6% (38)
Not sure or prefer not to answer	5.0% (5)
**Health insurance**	
Yes	98.0% (99)
No	1.0% (1)
Not sure/prefer not to answer	1.0% (1)

### Preference weights

**Fig. 2.**
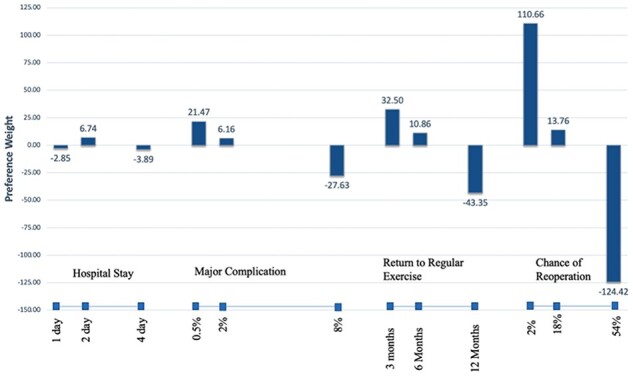
Shows the preference weight estimates for each of the intra-attribute levels. Attribute levels may be compared with other levels within the attribute but cannot be compared with levels of separate attributes. It is important to note that preference weights for levels within attributes are not linear; this indicates different utilities at difference levels and indicates excellent respondent attention and instrument design. For example, respondents tolerate a month of exercise limitations more in the short term than in the long term.

The results demonstrated face validity—estimated average preference weights were generally correlated with expected preference weights, where the most favorable attributes were weighted most heavily and the other weights followed in descending order (Fig. 2). All levels within attributes were found to be significantly different (*P* < 0.05) from one another except for 1 day versus 4 day hospital stay. The only levels within an attribute that were found to have a significant impact on the patient final choice via logistic regression were 6 Months to Resume Regular Exercise and 2% Chance Reoperation (*P* < 0.05).

### Overall attribute importance

**Fig. 3.**
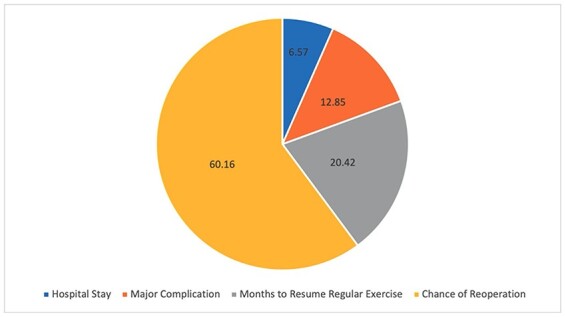
Displays the overall attribute importance from our data. Chance of Reoperation was overall the most important attribute, and was three times more important than the next closest attribute (Months to Resume Regular Exercise).

Overall attribute importance is presented as a portion of the total where the sum of all attribute importance weights is 100. Chance of Reoperation was the most important attribute, with an average importance of 60.16 (95% CI: 56.99–63.34). Hospital Stay was found to have the least importance (average importance 6.57, 95% CI: 5.73–7.41) (Fig. 3). Statistically significant differences were found between each attribute’s relative importance (*P* < 0.05).

### MAR

MAR for Chance of Reoperation and Months to Resume Regular Exercise was calculated at 2.11. This means that participants were willing to accept an additional 2.11% Chance of Reoperation for each 1 month decrease in Months to Resume Regular Exercise.

### Latent class analysis

**Fig. 4.**
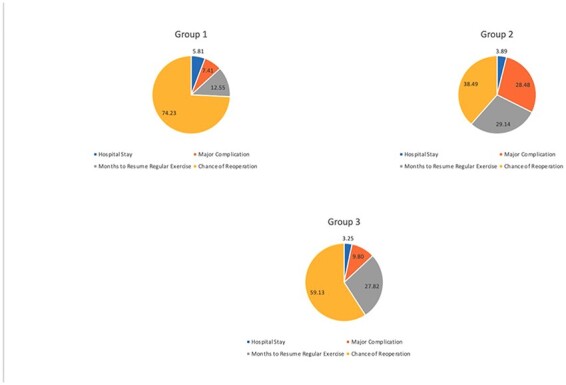
Displays the attribute importance stratified by latent class subgroup. Group 1 placed a much higher importance on Chance of Reoperation than either of the other groups; Groups 2 and 3 had a much more evenly distributed attribute importance profile. These factors help guide SDM conversations; Group 1 patients would most likely benefit from talking about treatment that minimizes risk of reoperation, while Group 2 and 3 patients would likely benefit from spending more time talking about risk–benefit tradeoffs for each surgical treatment option.

The Latent Class analysis was run for models including two to five subgroups. A three-subgroup model was found to have the best fit (LL = –404.3, BIC 1009.2). Overall trends in preference weights and importance weights for each group matched those reported for the entire cohort (Chance of Reoperation remained the most important attribute). Group 1 (53.0%) included the largest proportion of the sample, followed by Group 3 (25.5%) and Group 2 (21.6%). Group 1 had the highest importance weight for Chance of Reoperation (74.2) and lowest importance weight for Months to Resume Regular Exercise (12.6). Group 2 had the lowest weight for Chance of Reoperation (38.5). Groups 2 and 3 had similar importance weights for Months to Resume Regular Exercise (29.1, 27.8) (Fig. 4).

### Final choice

The final choice question discerning patient preference for PAO and arthroscopy versus arthroscopy alone yielded an almost even split. A total of 51 patients (51.5%) selected PAO and arthroscopy, while 50 (49.5%) selected arthroscopy alone. The average percent certainty from participants on their decision was 68.8% ± 23.0% (Table II).

## DISCUSSION

This study supports our hypothesis that decision-making for surgical treatment of FAIS with BHD is highly sensitive to patient preferences. Notably, preferences between the two surgeries were split almost evenly. When considering surgical treatment for their hip condition, we found that 2% Chance of Reoperation within 2 years as well as 6 Months to Resume Regular Exercise were the only levels of attributes to have significant impact on the patient’s ultimate choice. We believe that patient decision-making is driven by a desire to minimize their risk while balancing the quickest return to normal life. Thus, patients tended to opt for the lowest reoperation risk (2% Chance of Reoperation). However, their preference for a 6-month Return to Regular Exercise suggest that 3 months would be too short (and thus a greater risk of reinjury), while 12 months was too long.

The Latent Class analysis explored if participants can be grouped based on similarities in the weights they assign to attributes and their respective levels. Roughly half of our sample (Group 1) highly valued avoiding another operation, and timing to resume regular exercise was much less important when compared with the other groups. In comparison, Group 2 and Group 3 had more distributed preferences; both groups valued Chance of Reoperation less than Group 1 but valued other attributes like Major Complication risk and Time to Resume Regular Exercise more. These participants would likely benefit from a provider-led discussion with emphasis on the specifics surrounding risk–benefit tradeoffs to inform their treatment preference, highlighting the importance of SDM.

SDM is best defined as the overlap between what patients are experts in (their preferences, values and expectations) and what clinicians are experts in (medical evidence); optimal SDM requires knowledge of both topics. This study informs several aspects of this framework. First, this treatment decision is highly preference sensitive, confirming the necessary role of the SDM process. Second, it streamlines that process to inform clinicians on attributes that are important to most patients (Chance of Reoperation) and less important (Length of Hospital Stay) in the decision-making process. Third, tools used to identify these preferences can also serve as an educational instrument for patients, providing background information about their pathology, treatment and recovery process that can make SDM conversations more focused and beneficial. It should be noted that patient preferences are only a portion of the surgical decision-making process, and that other biomedical factors play an important role as well. The findings of this study are aimed to highlight the importance of the SDM. The actual findings of specific preferences as reported in this study should only be employed to make surgical decisions in clinical scenarios when there is truly no correct choice between arthroscopy alone and PAO + arthroscopy. Further studies are necessary to continue to explore how to match the correct procedure to each patient.

This study also informs risk–benefit trade-offs. Respondents reported equal utility of returning to exercise 1 month quicker with 2% increased reoperation risk. Thus, if one treatment (arthroscopy alone in this study with a return to regular exercise of 6 months) has a maximum incremental return to regular exercise of 6 fewer months (compared to PAO which was fixed at return in 12 months), but an incrementally higher risk of reoperation of greater than 12%, then it is likely that few, if any, patients would prefer the higher risk option. This last aspect of SDM highlights how the results of this study can inform future research and SDM instrument development. With Chance of Reoperation being the most important variable, future research should focus on accurately informing this risk for individual patients and then developing models and instruments to deliver this information to the patient and clinician at the point of care.

The latent class analysis results can facilitate SDM discussions by helping clinicians identify preference phenotypes. For example, patients in Class 1 have a very high importance on Chance of Reoperation. Their SDM conversations should focus on minimizing the risk of reoperation, generally favoring PAO. Patients in Class 2 and 3 have more distributed preferences; thus, their discussions should focus on risk–benefit tradeoffs. These methods offer a path toward enabling pragmatic delivery of individual patient preferences at the point of care. Surgeons may apply the knowledge from this study in recognizing that a significant proportion of patients would accept the hospital stay, risk of complication and increased return to sport associated with PAO in exchange for a lower reoperation rate. Some surgeons may view PAO as a significant undertaking; acknowledging that patients are accepting of the relative morbidity of PAO compared to arthroscopy in the setting of BHD may help surgeons overcome their own bias and procedural preferences.

This study included several limitations. Data collection was performed at only one academic medical center and thus may not be fully representative of the BHD population. Many participants were offered participation in the study via email or telephone prior to their presentation to clinic; yet, no response was received. We believe this low response rate was due to the additional burden the patients required to complete the survey on their own. Furthermore, IRB approval for this study was obtained as part of a larger study, and thus the inclusion criteria age range was broader than the typical hip preservation population. However, both the mean and median age of participants were under 40 years old, representative of a typical hip preservation population [56]. Our study population was 80% Caucasian and therefore these data may not be applicable to other borderline hip dysplasia populations. Attribute and level selection is limited by cognitive burden—thus, fewer attributes and levels lead to greater response accuracy. The results suggest an excellent level of accuracy illustrated by generally excellent quality responses and non-linearity of the utilities, but the limitation is that we may have not captured all relevant factors influencing the decision in the survey [57]. Future studies should incorporate factors like pain relief and amount of persistent pain, preoperative morbidity, length of incision/scar, time under anesthesia, post-operative pain, weightbearing status, rehabilitation, progression of osteoarthritis and other factors that may influence surgical treatment preference. Furthermore, CT scan with 3D reconstruction may also be helpful for discerning whether symptoms are being driven by an impingement versus instability morphology.

Probability of reoperation in this study was presented as an absolute risk over a defined time period (2 years). Indefinite cumulative rate of failure, such as 1% reoperation rate per year, may have impacted patient preference and decision-making. However, long-term outcome data following osteotomy and arthroscopy for FAIS in the setting of BHD, particularly using modern diagnostic and surgical approaches, remain lacking for providing accurate risk profiles. With the confines of the study design, reoperation theoretically would include a spectrum of procedures from hardware removal to labral reconstruction. Differences in morbidity of revision operative procedures may have contributed to patient preference. Patient baseline activity level, occupation and sporting choices may have also factored into their preference and may be an opportunity for future research. Furthermore, for each individual patient, we do not truly know the chance of reoperation or major complication, as these levels were taken as population estimates and are not specific to each patient taking the survey. Structural pathology of the hip comprises a broad spectrum of diagnosis ranging from hip instability to FAIS. Anatomical risk factors such as femoral and acetabular version, patholaxity and spinopelvic motion all represent distinct patient categories that may or may not benefit more from arthroscopy versus osteotomy. Attributing hip pain based on these demographic and anatomical risk factors is a tremendous opportunity for predictive modeling. In this study, the limitations of history, physical exam and radiographic parameters were used to isolate patients with FAIS in the setting of BHD. Reported reoperation rate, return to activity, hospital length of stay and complication profile may not apply to patients with underlying variable acetabular and femoral morphologies, and is a limitation of this study. Future studies utilizing predictive modeling specific to patient demographic and anatomic variables may more accurately estimate outcome and risk, and thus impact patient decision-making.

The relative importance of reoperation risk may also be inflated by the breadth of the attribute levels in this study. However, if such inflation occurred, its magnitude was likely insufficient to change the relative importance ranking of this attribute. Patient baseline understanding of borderline hip dysplasia was not accounted for in this study; although we provided basic education to each participant, increased understanding from factors such online education, counseling from other physicians and personal experience may have all imparted bias into patient preference.

Low enrollment numbers and statistical power could also be considered a flaw in this study. There are unclear standards on the necessary sample size needed for DCEs; minimal numbers of subjects have been stated at 20, 100 and 150 in previous literature [36, 58, 59]. However, power analysis done with previously published methods yielded the necessary number of patients to be 75 [60]. Lastly, the scenario of primary hip arthroscopy with revision to PAO or future arthroplasty in the event of failure remains a clinical consideration, but was not addressed in this study.

## CONCLUSION

When no clear surgical treatment is indicated, patient preferences have an amplified role in patient decision-making. Our results confirm variation in attribute importance within treatments as well as treatment choice, highlighting the importance in understanding patient preferences in decision-making for FAIS in BHD. More patient-specific generalizable outcomes of surgical treatment options are needed in the literature.

## Supplementary Material

hnae002_Supp

## Data Availability

The data underlying this article will be shared on reasonable request to the corresponding author.
