# Supplementary material for: Decision-making in borderline hip dysplasia and concomitant femoracetabular impingement syndrome: using a discrete choice experiment to explore patient preferences
Source: J Hip Preserv Surg. 2024 Feb 1;11(3):167–75. doi: 10.1093/jhps/hnae002 (PMC11631526; doi:10.1093/jhps/hnae002)
Supplement: hnae002_Supp [file hnae002_supp.zip › suppl_data/PAO Scope Addendum_PreSurg Counseling_JHP.docx]

Addendum: Discussion Points Used in Pre-Surgical Counseling

- History taking: understanding if symptoms stemmed more from their dysplasia (instability) or from impingement? Instability would lead us to considering a combined PAO/scope, whereas symptoms driven by impingement would lead us to recommend arthroscopy alone.
- Desired Activity Level: to what activity level to patients wish to return? What is their hip preventing them from doing? This was important to understand what dynamic positions the patient would be putting their hip through in the future; patients seeking to return to low impact, low stress activities may not see increased benefit from the additional bony stability that a PAO provides when compared to hip arthroscopy.
- Surgical Preferences: How quickly does the patient wish to return to sport? Are they agreeable to taking on increased risk of additional surgery in exchange for a quicker recovery? Does time in the hospital affect their decision-making? These factors that we explore with our discrete choice experiment are an important part of our surgical decision-making process with the patient.
